# Author Correction: Integrative single-cell characterization of a frugivorous and an insectivorous bat kidney and pancreas

**DOI:** 10.1038/s41467-024-44937-5

**Published:** 2024-02-27

**Authors:** Wei E. Gordon, Seungbyn Baek, Hai P. Nguyen, Yien-Ming Kuo, Rachael Bradley, Sarah L. Fong, Nayeon Kim, Alex Galazyuk, Insuk Lee, Melissa R. Ingala, Nancy B. Simmons, Tony Schountz, Lisa Noelle Cooper, Ilias Georgakopoulos-Soares, Martin Hemberg, Nadav Ahituv

**Affiliations:** 1 Department of Bioengineering and Therapeutic Sciences, University of California, San Francisco, San Francisco, CA 94158 USA; 2 Institute for Human Genetics, University of California, San Francisco, San Francisco, CA 94158 USA; 3 Department of Biotechnology, College of Life Science and Biotechnology, Yonsei University, Seoul, 03722 Republic of Korea; 4 Department of Ophthalmology, University of California, San Francisco, San Francisco, CA 94143 USA; 5 Hearing Research Focus Area, Department of Anatomy and Neurobiology, Northeast Ohio Medical University, Rootstown, OH USA; 6 POSTECH Biotech Center, Pohang University of Science and Technology (POSTECH), Pohang, 37673 Republic of Korea; 7 Department of Biological Sciences, Fairleigh Dickinson University, Madison, NJ 07940 USA; 8 Division of Vertebrate Zoology, Department of Mammalogy, American Museum of Natural History, New York, NY 10024 USA; 9 Department of Microbiology, Immunology, and Pathology, College of Veterinary Medicine and Biomedical Sciences, Colorado State University, Fort Collins, CO 80523 USA; 10 Musculoskeletal Research Focus Area, Department of Anatomy and Neurobiology, Northeast Ohio Medical University, Rootstown, OH 44272 USA; 11 Institute for Personalized Medicine, Department of Biochemistry and Molecular Biology, The Pennsylvania State University College of Medicine, Hershey, PA 17033 USA; 12 Gene Lay Institute of Immunology and Inflammation, Brigham and Women’s Hospital and Harvard Medical School, Boston, MA 02115 USA; 13 Present Address: Department of Biology, Menlo College, 1000 El Camino Real, Atherton, CA 94027 USA

**Keywords:** Gene regulation, Evolutionary genetics, Genomics

Correction to: *Nature Communications* 10.1038/s41467-023-44186-y, published online 09 January 2024

The original version of this Article contained an error in Fig. 6. In the original version of Fig. 6, the arrow under “gene expression” for big brown bat “urine-concentration” was red and pointing down when it should have been green and pointing up. This has been corrected in both the PDF and HTML versions of the Article.

