# Supplementary material for: Integrative single-cell characterization of a frugivorous and an insectivorous bat kidney and pancreas
Source: Nat Commun. 2024 Jan 9;15:12. doi: 10.1038/s41467-023-44186-y (PMC10776631; doi:10.1038/s41467-023-44186-y)
Supplement: Supplementary file 1 — Supplementary Information [file 41467_2023_44186_MOESM1_ESM.pdf]

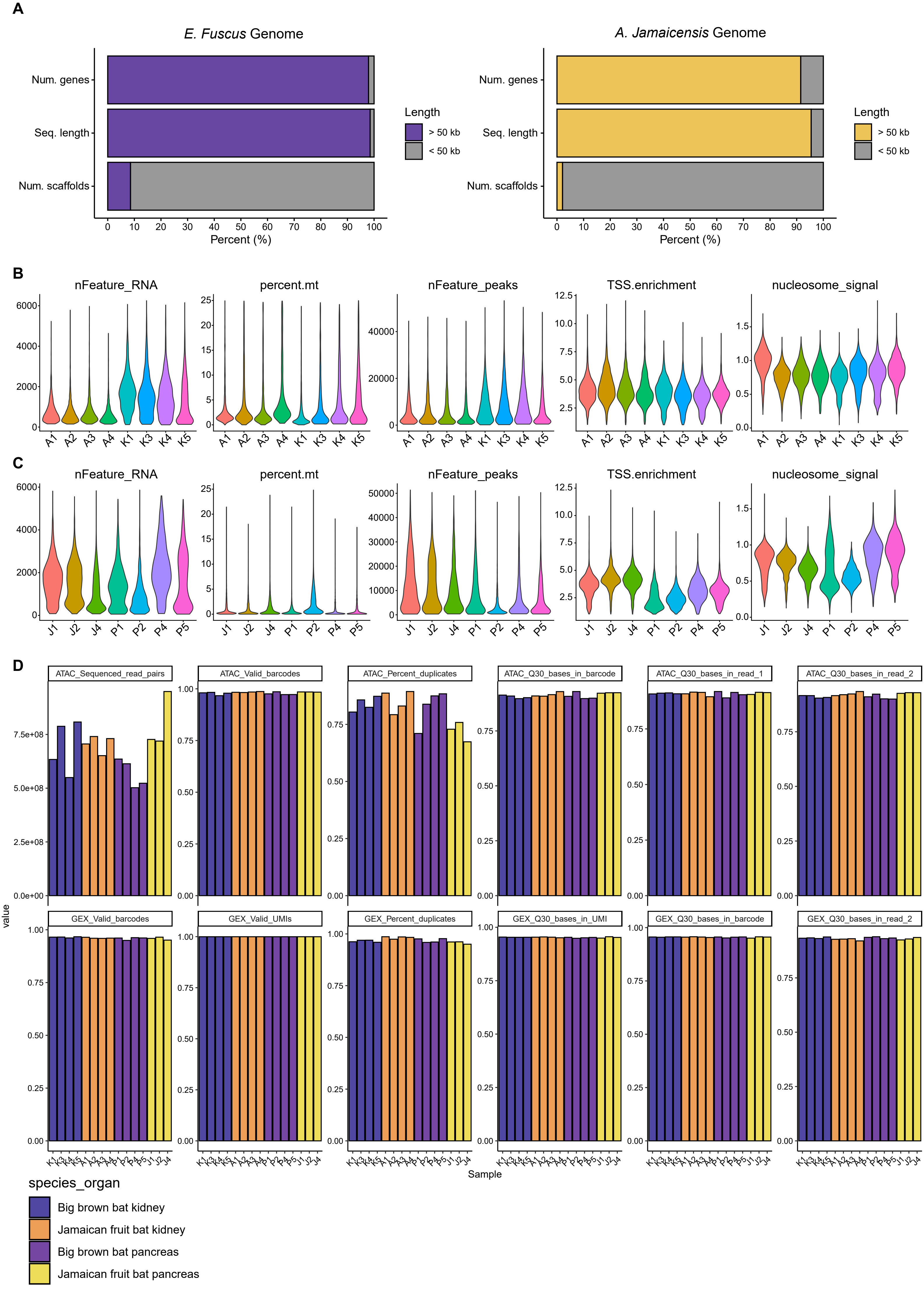

A
E. Fuscus Genome
A. Jamaicensis Genome
Num. genes
Seq. length
Num. scaffolds
Percent (%)
Length
> 50 kb
< 50 kb
B
C
nFeature_RNA
percent.mt
nFeature_peaks
TSS.enrichment
nucleosome_signal
D
ATAC_Sequenced_read_pairs
ATAC_Valid_barcodes
ATAC_Percent_duplicates
ATAC_Q30_bases_in_barcode
ATAC_Q30_bases_in_read_1
ATAC_Q30_bases_in_read_2
GEX_Valid_barcodes
GEX_Valid_UMIs
GEX_Percent_duplicates
GEX_Q30_bases_in_UMI
GEX_Q30_bases_in_barcode
GEX_Q30_bases_in_read_2
value
Sample
species_organ
Big brown bat kidney
Jamaican fruit bat kidney
Big brown bat pancreas
Jamaican fruit bat pancreas

Supplementary Fig.1: Joint scRNA and scATAC design and processing in bat tissues.

a, Bar chart showing that the number of genes and the genome sequence length captured by the number of scaffolds > 50 kb is > 90% for both bat genomes used in this study. **b-d,** QC metrics for single-cell multiome on bat kidneys and pancreases. A1-2 = fasted Jamaican fruit (JF) bat kidneys, A3-4 = treated Jamaican fruit (JF) bat kidneys, K1-3 = treated big brown (BB) bat kidneys, K4-5 = fasted big brown (BB) bat kidneys, J1-2 = fasted Jamaican fruit (JF) bat pancreases, J4= treated Jamaican fruit (JF) bat pancreas, P1-2 = treated big brown (BB) bat pancreases, P4-5 = fasted big brown (BB) bat pancreases. **b-c)** Violin plots of QC metrics Num. RNA features (nFeature_RNA), mitochondrial percentage (percent.mt), Num. ATAC features (nFeature_peaks), transcription start site enrichment (TSS.enrichment), and nucleosome signal **d,** Bar plots of QC metrics in **Supplementary Data 1**.

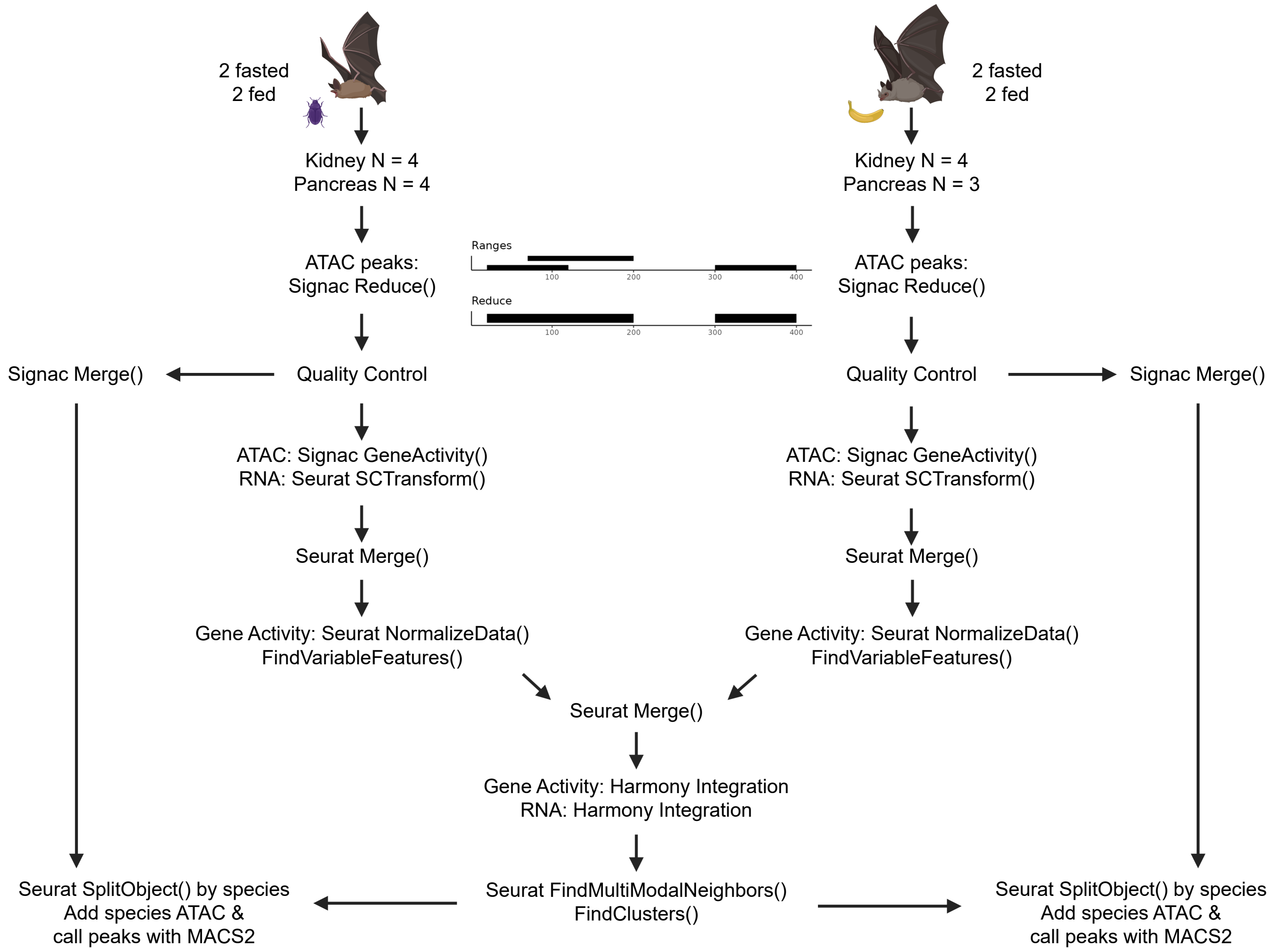
2 fasted
2 fed
Kidney N = 4
Pancreas N = 4
ATAC peaks:
Signac Reduce()
Quality Control
Signac Merge()
ATAC: Signac GeneActivity()
RNA: Seurat SCTransform()
Seurat Merge()
Gene Activity: Seurat NormalizeData()
FindVariableFeatures()
2 fasted
2 fed
Kidney N = 4
Pancreas N = 3
ATAC peaks:
Signac Reduce()
Quality Control
Signac Merge()
ATAC: Signac GeneActivity()
RNA: Seurat SCTransform()
Seurat Merge()
Gene Activity: Seurat NormalizeData()
FindVariableFeatures()
Ranges
100 200 300 400
Reduce
100 200 300 400
Seurat Merge()
Gene Activity: Harmony Integration
RNA: Harmony Integration
Seurat FindMultiModalNeighbors()
FindClusters()
Seurat SplitObject() by species
Add species ATAC &
call peaks with MACS2
Seurat SplitObject() by species
Add species ATAC &
call peaks with MACS2

Supplementary Fig.2: Schematic for cross species integration of multi-omic data. *E. fuscus* denoted by big brown (BB) bat on the left, and *A. jamaicensis* denoted by Jamaican fruit (JF) bat on the right (ranges and reduce image adapted from Signac[1]; created with BioRender.com).

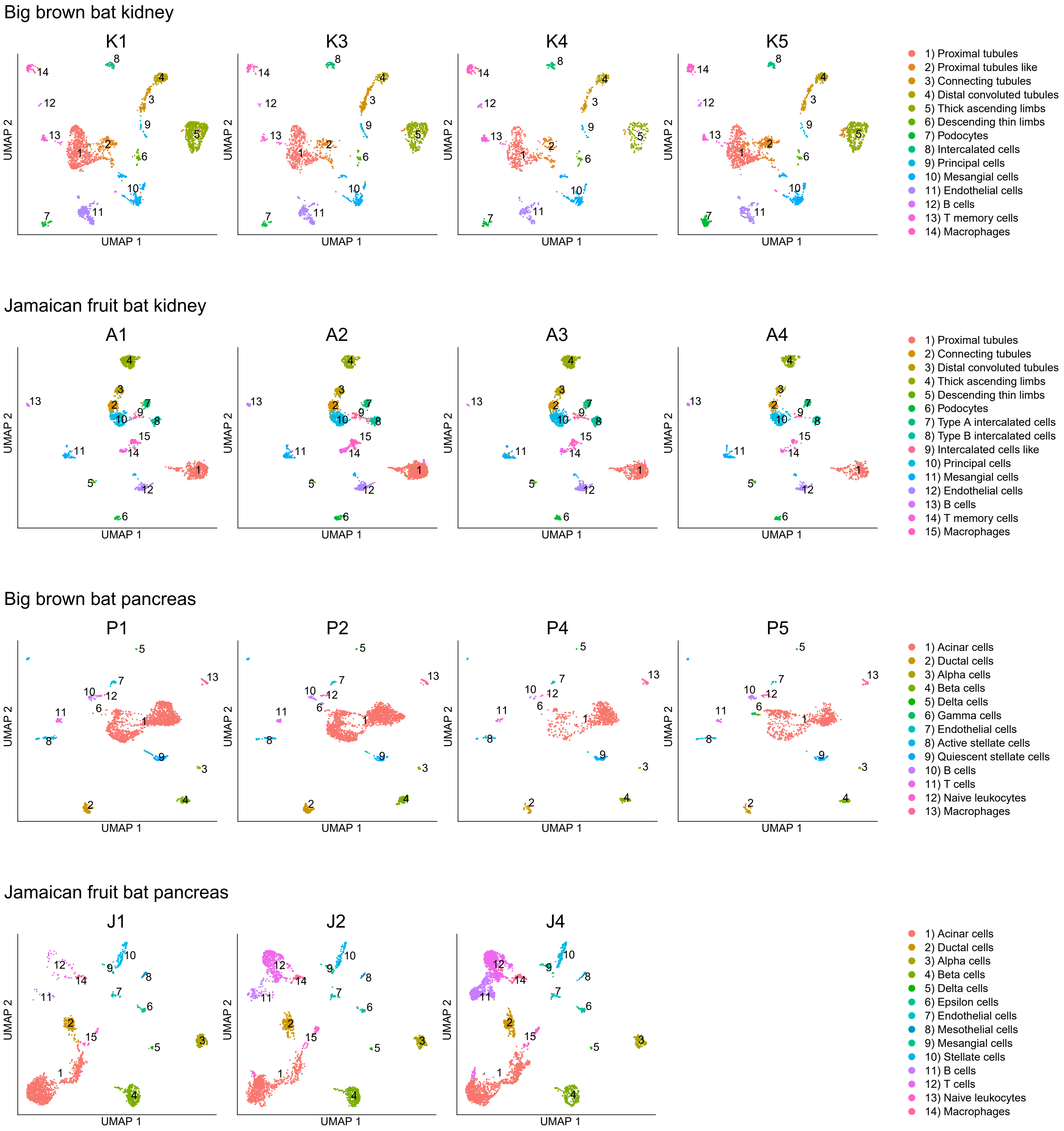

Big brown bat kidney
K1
K3
K4
K5
UMAP 1
UMAP 2
1) Proximal tubules
2) Proximal tubules like
3) Connecting tubules
4) Distal convoluted tubules
5) Thick ascending limbs
6) Descending thin limbs
7) Podocytes
8) Intercalated cells
9) Principal cells
10) Mesangial cells
11) Endothelial cells
12) B cells
13) T memory cells
14) Macrophages
Jamaican fruit bat kidney
A1
A2
A3
A4
1) Proximal tubules
2) Connecting tubules
3) Distal convoluted tubules
4) Thick ascending limbs
5) Descending thin limbs
6) Podocytes
7) Type A intercalated cells
8) Type B intercalated cells
9) Intercalated cells like
10) Principal cells
11) Mesangial cells
12) Endothelial cells
13) B cells
14) T memory cells
15) Macrophages
Big brown bat pancreas
P1
P2
P4
P5
1) Acinar cells
2) Ductal cells
3) Alpha cells
4) Beta cells
5) Delta cells
6) Gamma cells
7) Endothelial cells
8) Active stellate cells
9) Quiescent stellate cells
10) B cells
11) T cells
12) Naive leukocytes
13) Macrophages
Jamaican fruit bat pancreas
J1
J2
J4
1) Acinar cells
2) Ductal cells
3) Alpha cells
4) Beta cells
5) Delta cells
6) Epsilon cells
7) Endothelial cells
8) Mesothelial cells
9) Mesangial cells
10) Stellate cells
11) B cells
12) T cells
13) Naive leukocytes
14) Macrophages

Supplementary Fig.3: Cell-type annotations across samples within each tissue of each species.

K1-3 = treated big brown (BB) bat kidneys, K4-5 = fasted big brown (BB) bat kidneys, A1-2 = fasted Jamaican fruit (JF) bat kidneys, A3-4 = treated Jamaican fruit (JF) bat kidneys, P1-2 = treated big brown (BB) bat pancreases, P4-5 = fasted big brown (BB) bat pancreases, J1-2 = fasted Jamaican fruit (JF) bat pancreases, J4= treated Jamaican fruit (JF) bat pancreas.

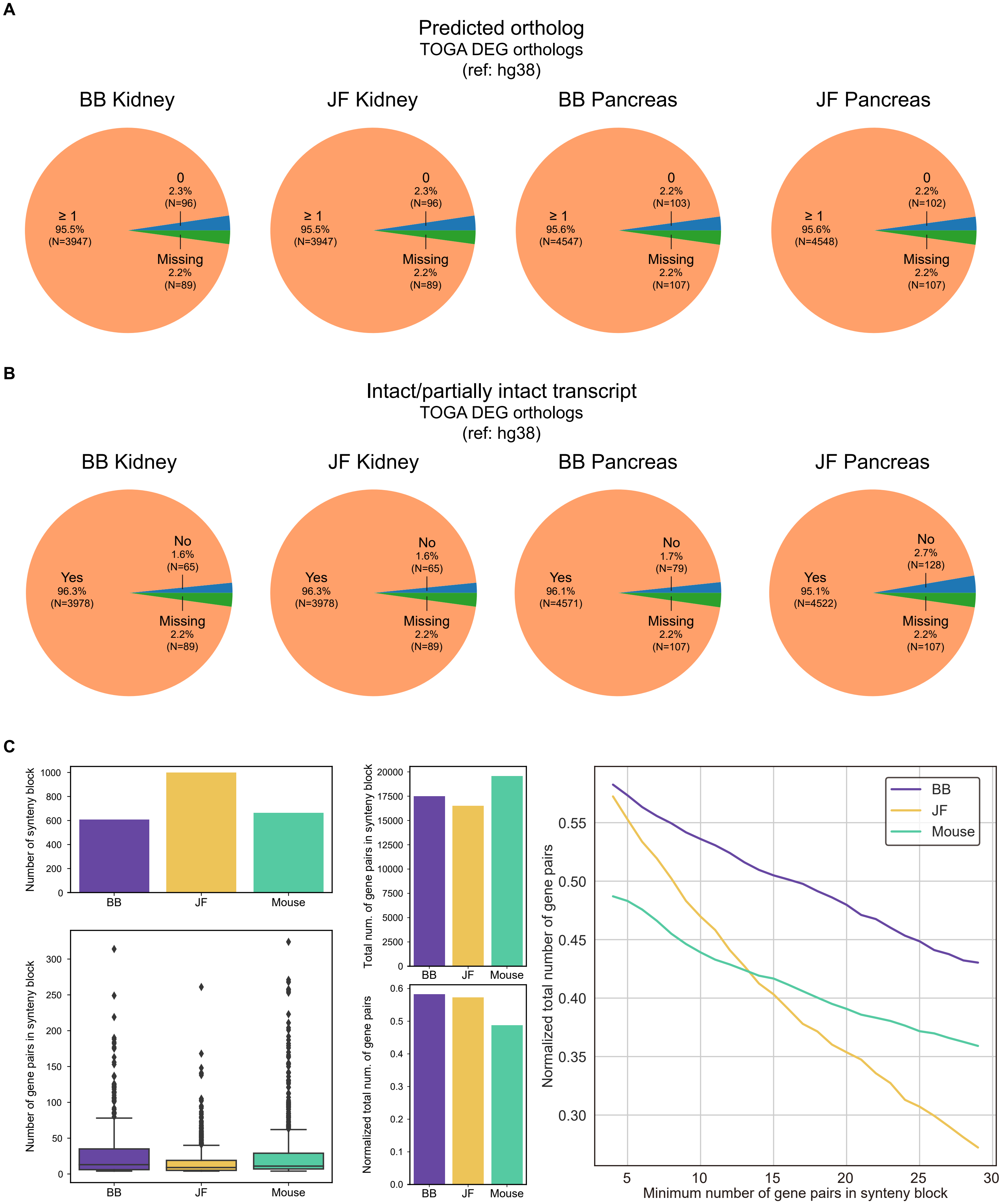
A
Predicted ortholog
TOGA DEG orthologs
(ref: hg38)
BB Kidney
0
2.3%
(N=96)
≥ 1
95.5%
(N=3947)
Missing
2.2%
(N=89)
JF Kidney
0
2.3%
(N=96)
≥ 1
95.5%
(N=3947)
Missing
2.2%
(N=89)
BB Pancreas
0
2.2%
(N=103)
≥ 1
95.6%
(N=4547)
Missing
2.2%
(N=107)
JF Pancreas
0
2.2%
(N=102)
≥ 1
95.6%
(N=4548)
Missing
2.2%
(N=107)
B
Intact/partially intact transcript
TOGA DEG orthologs
(ref: hg38)
BB Kidney
No
1.6%
(N=65)
Yes
96.3%
(N=3978)
Missing
2.2%
(N=89)
JF Kidney
No
1.6%
(N=65)
Yes
96.3%
(N=3978)
Missing
2.2%
(N=89)
BB Pancreas
No
1.7%
(N=79)
Yes
96.1%
(N=4571)
Missing
2.2%
(N=107)
JF Pancreas
No
2.7%
(N=128)
Yes
95.1%
(N=4522)
Missing
2.2%
(N=107)
C
Number of synteny block
BB
JF
Mouse
Number of gene pairs in synteny block
Total num. of gene pairs in synteny block
Normalized total num. of gene pairs
Normalized total number of gene pairs
Minimum number of gene pairs in synteny block
BB
JF
Mouse

Supplementary Fig.4: Evaluation of gene orthology and synteny between bats and humans.

a, Number of differentially expressed genes between bats in each tissue that were predicted by TOGA[2] to have ≥1 human orthologs, 0 human orthologs, or no prediction ("Missing"). b, Number of differentially expressed genes between bats in each tissue that were predicted by TOGA[2] to have at least one human orthologous transcript that is intact or partially intact ("Yes"), not intact or partially intact ("No"), or no prediction ("Missing"). c, (Left) Number of synteny blocks and number of gene pairs in synteny blocks between each genome and human detected by MCscan[3] (see Methods). (Middle) Total number of gene pairs in synteny blocks and normalized total number of gene pairs in synteny blocks. (Right) Normalized total number of gene pairs by minimum number of gene pairs in synteny blocks. Jamaican fruit bat is depicted as JF and big brown bat as BB in the various panels. Source data are provided as a **Source Data** file.

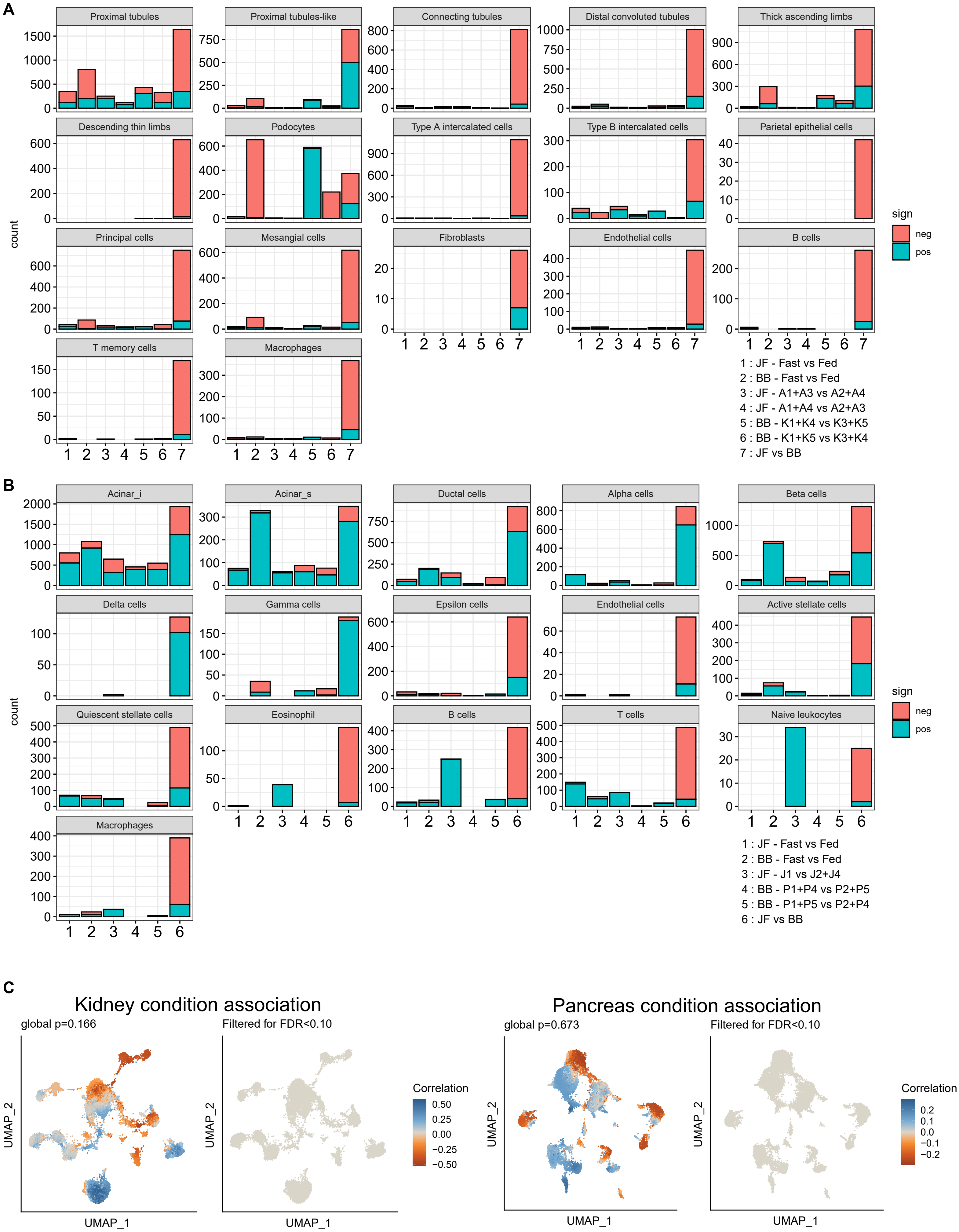
A
Proximal tubules
Proximal tubules-like
Connecting tubules
Distal convoluted tubules
Thick ascending limbs
Descending thin limbs
Podocytes
Type A intercalated cells
Type B intercalated cells
Parietal epithelial cells
Principal cells
Mesangial cells
Fibroblasts
Endothelial cells
B cells
T memory cells
Macrophages
count
sign
neg
pos
1 : JF - Fast vs Fed
2 : BB - Fast vs Fed
3 : JF - A1+A3 vs A2+A4
4 : JF - A1+A4 vs A2+A3
5 : BB - K1+K4 vs K3+K5
6 : BB - K1+K5 vs K3+K4
7 : JF vs BB
B
Acinar_i
Acinar_s
Ductal cells
Alpha cells
Beta cells
Delta cells
Gamma cells
Epsilon cells
Endothelial cells
Active stellate cells
Quiescent stellate cells
Eosinophil
B cells
T cells
Naive leukocytes
Macrophages
count
sign
neg
pos
1 : JF - Fast vs Fed
2 : BB - Fast vs Fed
3 : JF - J1 vs J2+J4
4 : BB - P1+P4 vs P2+P5
5 : BB - P1+P5 vs P2+P4
6 : JF vs BB
C
Kidney condition association
global p=0.166
Filtered for FDR<0.10
Correlation
0.50
0.25
0.00
−0.25
−0.50
Pancreas condition association
global p=0.673
Filtered for FDR<0.10
Correlation
0.2
0.1
0.0
−0.1
−0.2
UMAP_1
UMAP_2

Supplementary Fig.5: Differential expressed gene (DEG) counts by condition and by species in bat kidney and pancreas.

a, Bar charts of DEG counts in bat kidneys (legend notation: pos. vs neg.). A1-2 = fasted Jamaican fruit (JF) bats, A3-4 = treated Jamaican fruit (JF) bats, K1-3 = treated big brown (BB) bats, K4-5 = fasted big brown (BB) bats. (B) Bar charts of DEG counts in bat pancreases (legend notation: pos. vs neg.). J1-2 = fasted Jamaican fruit (JF) bats, J4= treated Jamaican fruit (JF) bats, P1-2 = treated big brown (BB) bats, P4-5 = fasted big brown (BB) bats. (C) (Left) CNA of condition associations to cell-types in bat kidney. (Right) CNA of condition associations to cell-types in bat pancreas. Global *p*-value calculated with permutation in CNA package. Jamaican fruit bat is depicted as JF and big brown bat as BB in the various panels.

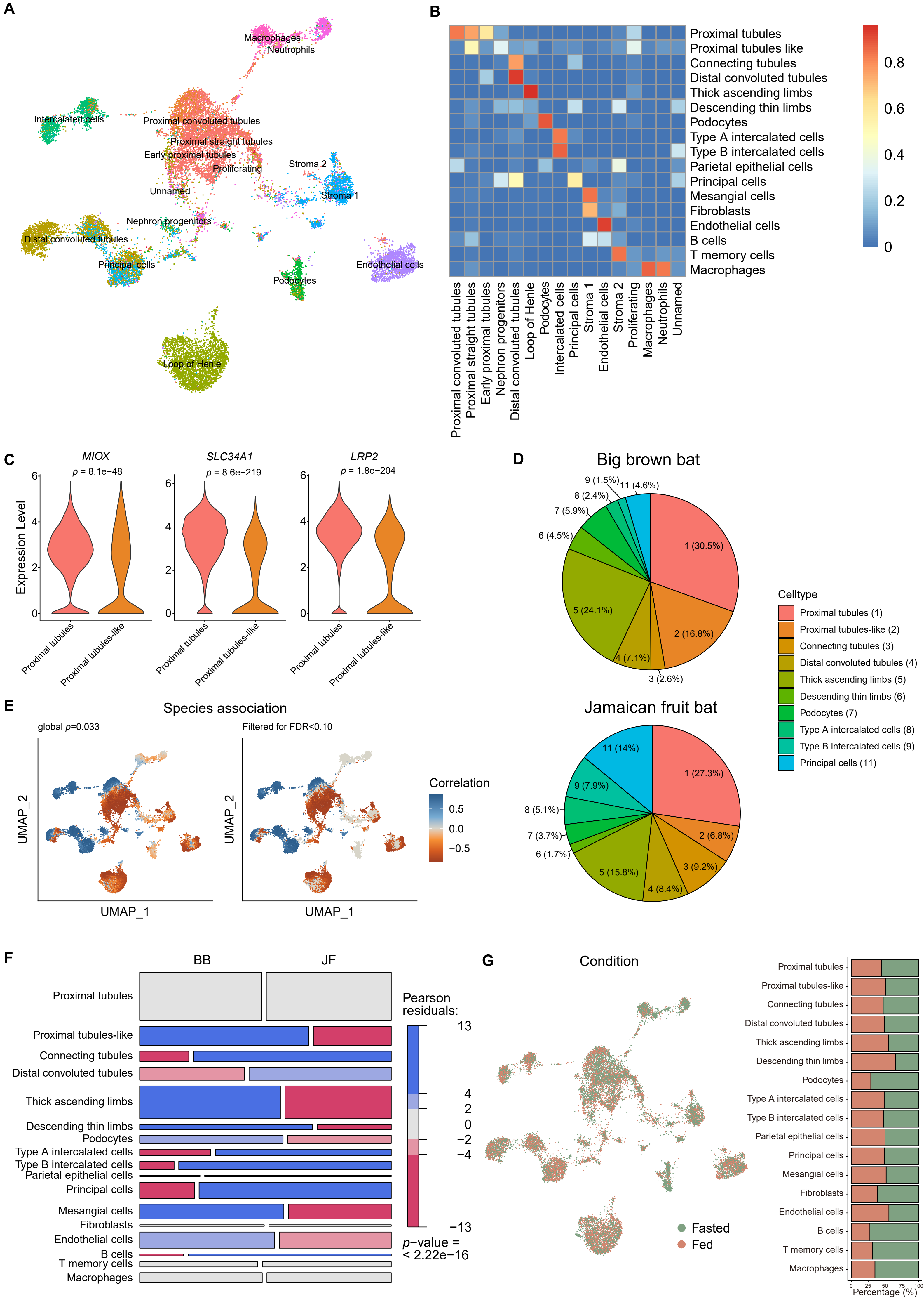

A
Macrophages
Neutrophils
Intercalated cells
Proximal convoluted tubules
Proximal straight tubules
Early proximal tubules
Proliferating
Stroma 2
Stroma 1
Unnamed
Nephron progenitors
Distal convoluted tubules
Principal cells
Endothelial cells
Podocytes
Loop of Henle
B
Proximal tubules
Proximal tubules like
Connecting tubules
Distal convoluted tubules
Thick ascending limbs
Descending thin limbs
Podocytes
Type A intercalated cells
Type B intercalated cells
Parietal epithelial cells
Principal cells
Mesangial cells
Fibroblasts
Endothelial cells
B cells
T memory cells
Macrophages
0.8
0.6
0.4
0.2
0
Proximal convoluted tubules
Proximal straight tubules
Early proximal tubules
Nephron progenitors
Distal convoluted tubules
Loop of Henle
Podocytes
Intercalated cells
Principal cells
Stroma 1
Endothelial cells
Stroma 2
Proliferating
Macrophages
Neutrophils
Unnamed
C
MIOX
p = 8.1e−48
SLC34A1
p = 8.6e−219
LRP2
p = 1.8e−204
Expression Level
Proximal tubules
Proximal tubules-like
D
Big brown bat
1 (30.5%)
2 (16.8%)
3 (2.6%)
4 (7.1%)
5 (24.1%)
6 (4.5%)
7 (5.9%)
8 (2.4%)
9 (1.5%)
11 (4.6%)
Jamaican fruit bat
1 (27.3%)
2 (6.8%)
3 (9.2%)
4 (8.4%)
5 (15.8%)
6 (1.7%)
7 (3.7%)
8 (5.1%)
9 (7.9%)
11 (14%)
Celltype
Proximal tubules (1)
Proximal tubules-like (2)
Connecting tubules (3)
Distal convoluted tubules (4)
Thick ascending limbs (5)
Descending thin limbs (6)
Podocytes (7)
Type A intercalated cells (8)
Type B intercalated cells (9)
Principal cells (11)
E
Species association
global p=0.033
Filtered for FDR<0.10
UMAP_2
UMAP_1
Correlation
0.5
0.0
−0.5
F
BB
JF
Proximal tubules
Proximal tubules-like
Connecting tubules
Distal convoluted tubules
Thick ascending limbs
Descending thin limbs
Podocytes
Type A intercalated cells
Type B intercalated cells
Parietal epithelial cells
Principal cells
Mesangial cells
Fibroblasts
Endothelial cells
B cells
T memory cells
Macrophages
Pearson residuals:
13
4
2
0
−2
−4
−13
p−value = < 2.22e−16
G
Condition
Fasted
Fed
Proximal tubules
Proximal tubules-like
Connecting tubules
Distal convoluted tubules
Thick ascending limbs
Descending thin limbs
Podocytes
Type A intercalated cells
Type B intercalated cells
Parietal epithelial cells
Principal cells
Mesangial cells
Fibroblasts
Endothelial cells
B cells
T memory cells
Macrophages
0 25 50 75 100
Percentage (%)

Supplementary Fig.6: Single-cell composition analysis of bat kidney.

a, UMAP of bat kidney cell-types automatically annotated with mouse kidney single-cell reference data from Azimuth[4]. b, Overlap coefficients of auto-annotations in A (horizontal) with our integrated species annotations in Fig.1d (vertical). c, Violin plots of proximal tubule marker gene expression in bat proximal tubules and proximal tubules-like cells. *P*-values calculated with two-sided Wilcoxon rank-sum test and the Bonferroni correction. d, Pie charts of cell-type percentages across renal epithelial cells. e, CNA of species associations to cell-types. Global *p*-value calculated with permutation in CNA package. f, Pearson residuals visualized by mosaic plot of all cell-types identified in bat kidneys. *P*-value calculated with chi-square test. g, UMAP of bat kidney cell-types by condition. The bar chart shows the proportion of conditions for each cell-type. Jamaican fruit bat is depicted as JF and big brown bat as BB in the various panels.

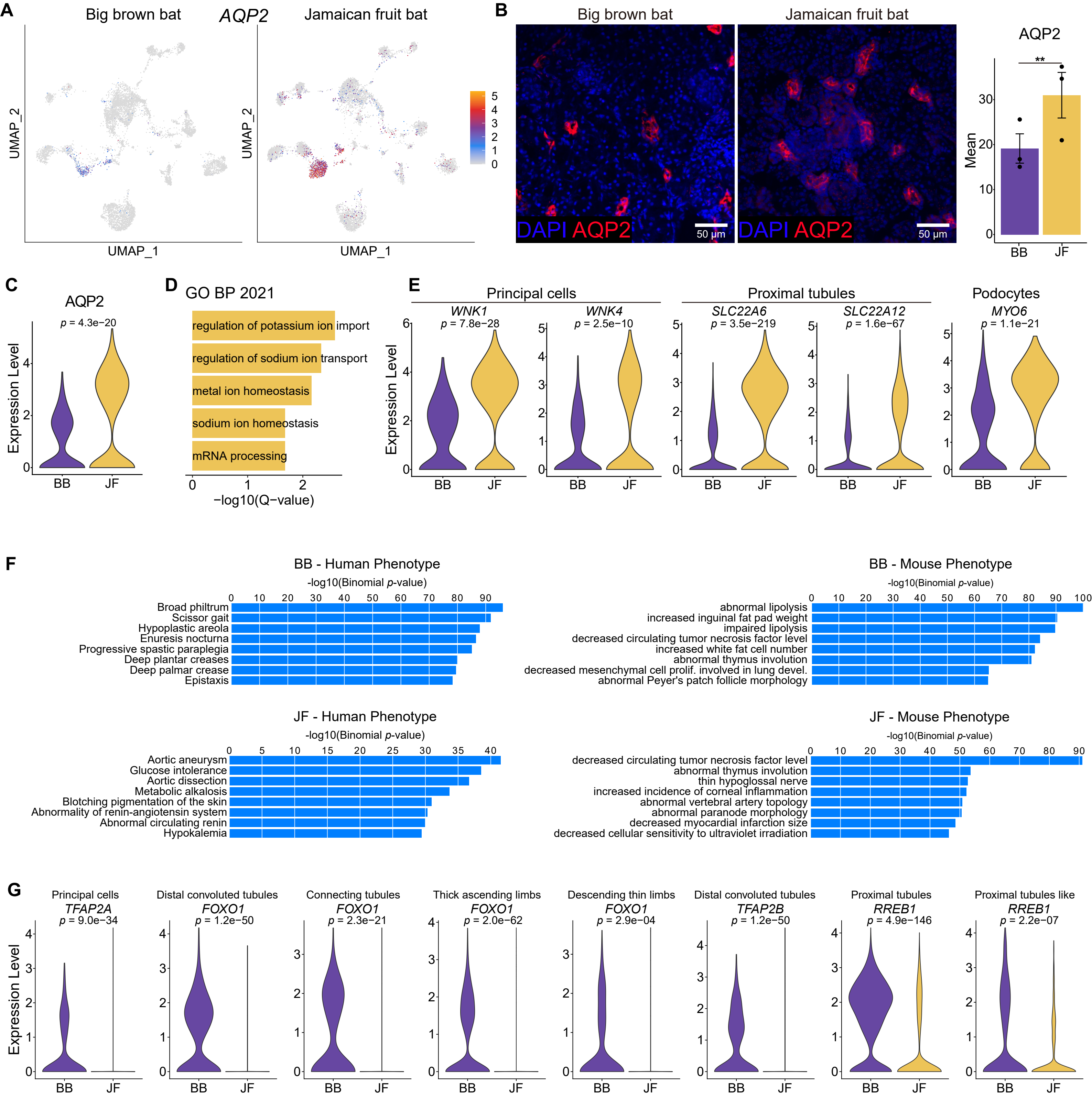
A
AQP2
Big brown bat
Jamaican fruit bat
UMAP_1
UMAP_2
B
DAPI AQP2
50 µm
AQP2
Mean
**
BB
JF
C
AQP2
p = 4.3e−20
Expression Level
D
GO BP 2021
regulation of potassium ion import
regulation of sodium ion transport
metal ion homeostasis
sodium ion homeostasis
mRNA processing
−log10(Q−value)
E
Principal cells
WNK1
p = 7.8e−28
WNK4
p = 2.5e−10
Proximal tubules
SLC22A6
p = 3.5e−219
SLC22A12
p = 1.6e−67
Podocytes
MYO6
p = 1.1e−21
F
BB - Human Phenotype
-log10(Binomial p-value)
Broad philtrum
Scissor gait
Hypoplastic areola
Enuresis nocturna
Progressive spastic paraplegia
Deep plantar creases
Deep palmar crease
Epistaxis
BB - Mouse Phenotype
abnormal lipolysis
increased inguinal fat pad weight
impaired lipolysis
decreased circulating tumor necrosis factor level
increased white fat cell number
abnormal thymus involution
decreased mesenchymal cell prolif. involved in lung devel.
abnormal Peyer's patch follicle morphology
JF - Human Phenotype
Aortic aneurysm
Glucose intolerance
Aortic dissection
Metabolic alkalosis
Blotching pigmentation of the skin
Abnormality of renin-angiotensin system
Abnormal circulating renin
Hypokalemia
JF - Mouse Phenotype
decreased circulating tumor necrosis factor level
abnormal thymus involution
thin hypoglossal nerve
increased incidence of corneal inflammation
abnormal vertebral artery topology
abnormal paranode morphology
decreased myocardial infarction size
decreased cellular sensitivity to ultraviolet irradiation
G
Principal cells
TFAP2A
p = 9.0e−34
Distal convoluted tubules
FOXO1
p = 1.2e−50
Connecting tubules
FOXO1
p = 2.3e−21
Thick ascending limbs
FOXO1
p = 2.0e−62
Descending thin limbs
FOXO1
p = 2.9e−04
Distal convoluted tubules
TFAP2B
p = 1.2e−50
Proximal tubules
RREB1
p = 4.9e−146
Proximal tubules like
RREB1
p = 2.2e−07

Supplementary Fig.7: scRNA-seq and scATAC-seq analysis of bat kidney.

a, UMAPs of principal cell marker gene *AQP2* expression in each species. b, (Left) Representative images of SLC26A4 immunofluorescence (red) in bat kidneys. Nuclei are stained with DAPI (blue). (Right) Quantification of AQP2 immunofluorescence normalized to nuclei in bat kidneys. Results represent arbitrary units of fluorescence (AU) mean ± standard error of the mean (SEM) derived from 3 big brown (BB) bats and 3 Jamaican fruit (JF) bats (n = 3/phenotype, n = 10 images/individual [see Methods]). Mixed effects model (two-sided) ***p*-value = .001. c, Violin plot of *AQP2* expression in principal cells. *P*-values calculated with two-sided Wilcoxon rank-sum test and the Bonferroni correction. d, Bar plots showing GO Biological Process 2021 terms enriched in Jamaican fruit (JF) bat principal cells. *Q*-values calculated with one-sided Fisher's exact test and corrected with the Benjamini-Hochberg method. e, Violin plots of differentially expressed genes in bat kidney cells. *P*-values calculated with two-sided Wilcoxon rank-sum test and the Bonferroni correction. f, Bar plots showing GREAT human and mouse phenotypes enriched in big brown (BB) bat and Jamaican fruit (JF) bat kidneys. g, Violin plots of differentially expressed TFs in bat renal epithelial cells. *P*-values calculated with two-sided Wilcoxon rank-sum test and the Bonferroni correction. Jamaican fruit bat is depicted as JF and big brown bat as BB in the various panels. Source data are provided as a **Source Data** file.

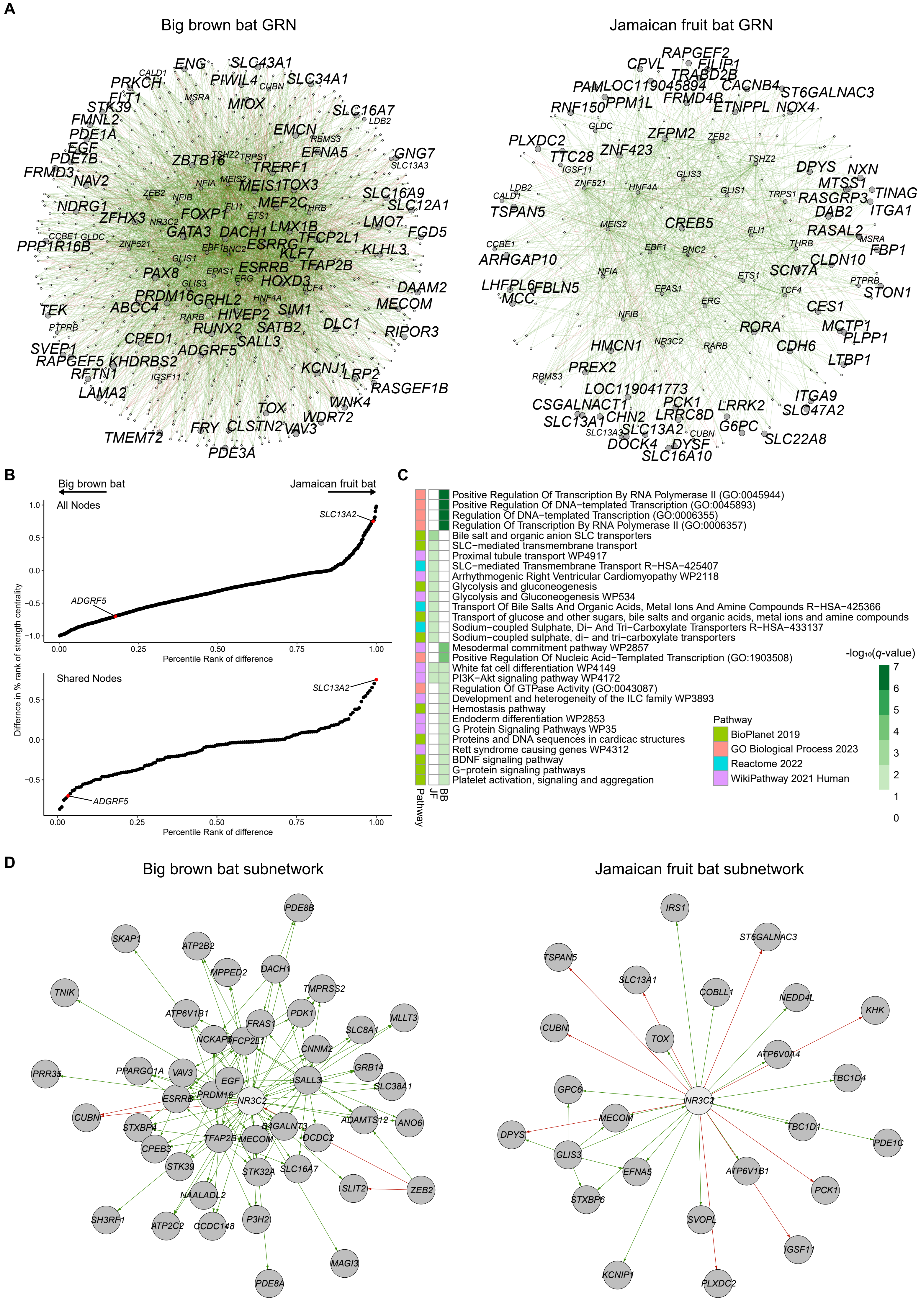
A
Big brown bat GRN
Jamaican fruit bat GRN
B
Big brown bat
Jamaican fruit bat
All Nodes
SLC13A2
ADGRF5
Percentile Rank of difference
Shared Nodes
Difference in % rank of strength centrality
C
Positive Regulation Of Transcription By RNA Polymerase II (GO:0045944)
Positive Regulation Of DNA−templated Transcription (GO:0045893)
Regulation Of DNA−templated Transcription (GO:0006355)
Regulation Of Transcription By RNA Polymerase II (GO:0006357)
Bile salt and organic anion SLC transporters
SLC−mediated transmembrane transport
Proximal tubule transport WP4917
SLC−mediated Transmembrane Transport R−HSA−425407
Arrhythmogenic Right Ventricular Cardiomyopathy WP2118
Glycolysis and gluconeogenesis
Glycolysis and Gluconeogenesis WP534
Transport Of Bile Salts And Organic Acids, Metal Ions And Amine Compounds R−HSA−425366
Transport of glucose and other sugars, bile salts and organic acids, metal ions and amine compounds
Sodium−coupled Sulphate, Di− And Tri−Carboxylate Transporters R−HSA−433137
Sodium−coupled sulphate, di− and tri−carboxylate transporters
Mesodermal commitment pathway WP2857
Positive Regulation Of Nucleic Acid−Templated Transcription (GO:1903508)
White fat cell differentiation WP4149
PI3K−Akt signaling pathway WP4172
Regulation Of GTPase Activity (GO:0043087)
Development and heterogeneity of the ILC family WP3893
Hemostasis pathway
Endoderm differentiation WP2853
G Protein Signaling Pathways WP35
Proteins and DNA sequences in cardicac structures
Rett syndrome causing genes WP4312
BDNF signaling pathway
G−protein signaling pathways
Platelet activation, signaling and aggregation
Pathway
JF
BB
Pathway
BioPlanet 2019
GO Biological Process 2023
Reactome 2022
WikiPathway 2021 Human
-log₁₀(q-value)
D
Big brown bat subnetwork
Jamaican fruit bat subnetwork
NR3C2

Supplementary Fig.8: Multi-omics GRN analyses of bat kidneys.

a, Big brown (BB) bat and Jamaican fruit (JF) bat GRNs determined by Pando[5]. b, Scatter plots of the percentile rank of species difference by the difference in percentile rank of strength centrality for all nodes (top) and shared nodes (bottom). c, Heatmap of enriched pathways in each species GRN. *Q*-values calculated with one-sided Fisher's exact test and corrected with the Benjamini-Hochberg method. d, *NR3C2* subnetworks in each species. Jamaican fruit bat is depicted as JF and big brown bat as BB in the various panels. Source data are provided as a **Source Data** file.

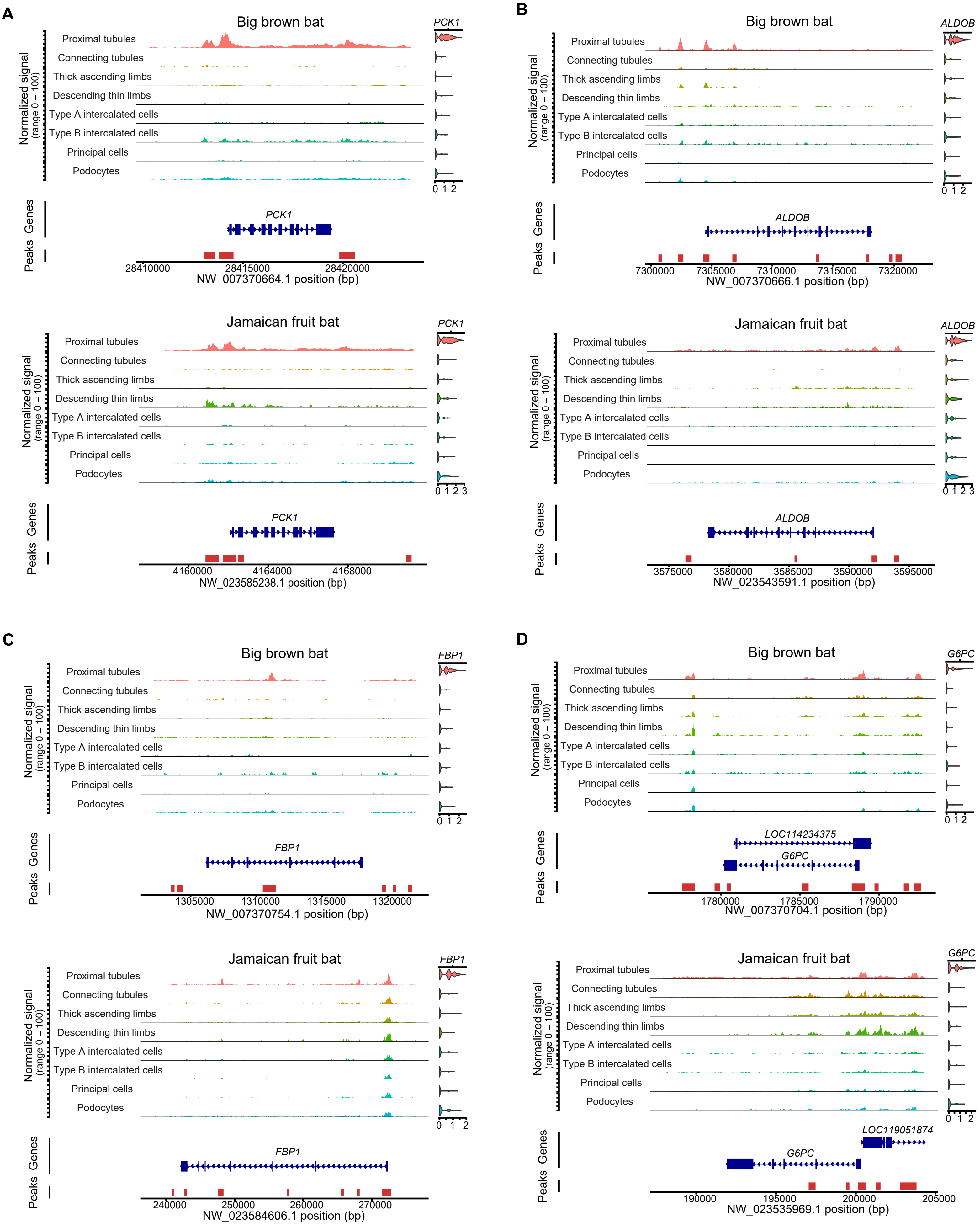
A
Big brown bat
PCK1
Normalized signal
(range 0 – 100)
Proximal tubules
Connecting tubules
Thick ascending limbs
Descending thin limbs
Type A intercalated cells
Type B intercalated cells
Principal cells
Podocytes
0 1 2
Genes
Peaks
PCK1
28410000
28415000
28420000
NW_007370664.1 position (bp)
Jamaican fruit bat
PCK1
0 1 2 3
PCK1
4160000
4164000
4168000
NW_023585238.1 position (bp)
B
Big brown bat
ALDOB
0 1 2
ALDOB
7300000
7305000
7310000
7315000
7320000
NW_007370666.1 position (bp)
Jamaican fruit bat
ALDOB
0 1 2 3
ALDOB
3575000
3580000
3585000
3590000
3595000
NW_023543591.1 position (bp)
C
Big brown bat
FBP1
0 1 2
FBP1
1305000
1310000
1315000
1320000
NW_007370754.1 position (bp)
Jamaican fruit bat
FBP1
0 1 2
FBP1
240000
250000
260000
270000
NW_023584606.1 position (bp)
D
Big brown bat
G6PC
0 1 2
LOC114234375
G6PC
1780000
1785000
1790000
NW_007370704.1 position (bp)
Jamaican fruit bat
G6PC
0 1 2
LOC119051874
G6PC
190000
195000
200000
205000
NW_023535969.1 position (bp)

Supplementary Fig.9: scATAC-seq coverage plots of diabetes-associated genes in bat kidneys.

a-d, scATAC-seq coverage plots of *PCK1* (a), *ALDOB* (b), *FBP1* (b) and *G6PC* (d) in bat kidneys. SCTransform-normalized expression plot visualized on the right by cell-type.

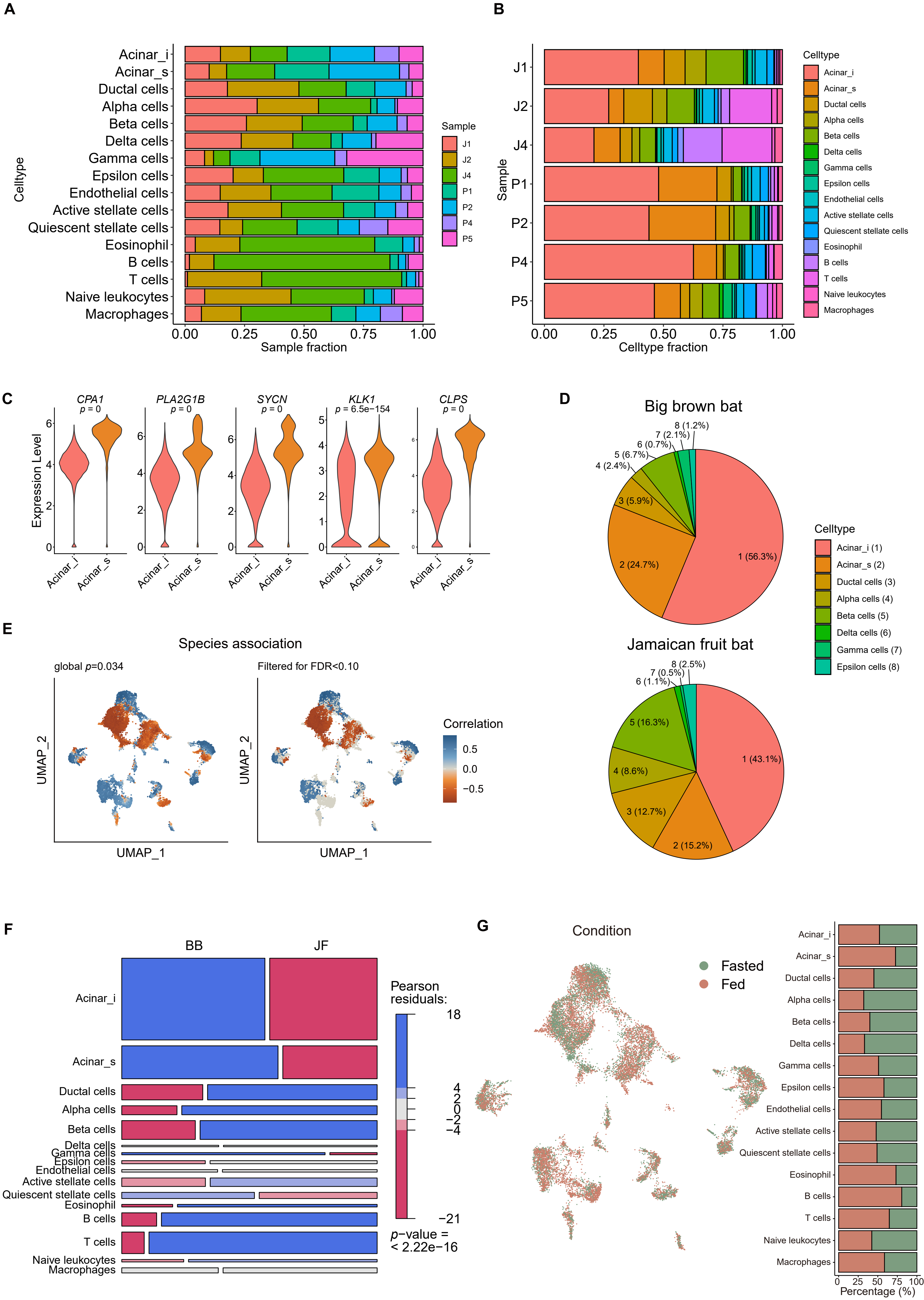

A
B
C
D
E
F
G
Sample fraction
Celltype fraction
Celltype
Sample
Expression Level
Big brown bat
Jamaican fruit bat
Species association
global p=0.034
Filtered for FDR<0.10
Correlation
UMAP_1
UMAP_2
BB
JF
Pearson residuals:
p−value = < 2.22e−16
Condition
Fasted
Fed
Percentage (%)
Acinar_i
Acinar_s
Ductal cells
Alpha cells
Beta cells
Delta cells
Gamma cells
Epsilon cells
Endothelial cells
Active stellate cells
Quiescent stellate cells
Eosinophil
B cells
T cells
Naive leukocytes
Macrophages
J1
J2
J4
P1
P2
P4
P5
CPA1
PLA2G1B
SYCN
KLK1
CLPS
p = 0
p = 6.5e−154
Acinar_i (1)
Acinar_s (2)
Ductal cells (3)
Alpha cells (4)
Beta cells (5)
Delta cells (6)
Gamma cells (7)
Epsilon cells (8)
1 (56.3%)
2 (24.7%)
3 (5.9%)
4 (2.4%)
5 (6.7%)
6 (0.7%)
7 (2.1%)
8 (1.2%)
1 (43.1%)
2 (15.2%)
3 (12.7%)
4 (8.6%)
5 (16.3%)
6 (1.1%)
7 (0.5%)
8 (2.5%)

Supplementary Fig.10: Single-cell composition analysis of bat pancreas.

a-b, a) Sample fraction across cell-type and b) Cell-type fraction across samples. J1-2 = fasted Jamaican fruit (JF) bats, J4= treated Jamaican fruit (JF) bat, P1-2 = treated big brown (BB) bats, P4-5 = fasted big brown (BB) bats. c, Violin plots of proximal tubule marker gene expression in bat proximal tubules and proximal tubules-like cells. d, Pie charts of cell-type percentages across renal epithelial cells. e, CNA of species associations to cell-types. Global *p*-value calculated with permutation in CNA package. f, Pearson residuals visualized by mosaic plot of all cell-types identified in bat pancreases. *P*-value calculated with chi-square test. g, UMAP of bat pancreases cell-types by condition. Bar chart shows the proportion per condition for each cell-type. Jamaican fruit bat is depicted as JF and big brown bat as BB in the various panels.

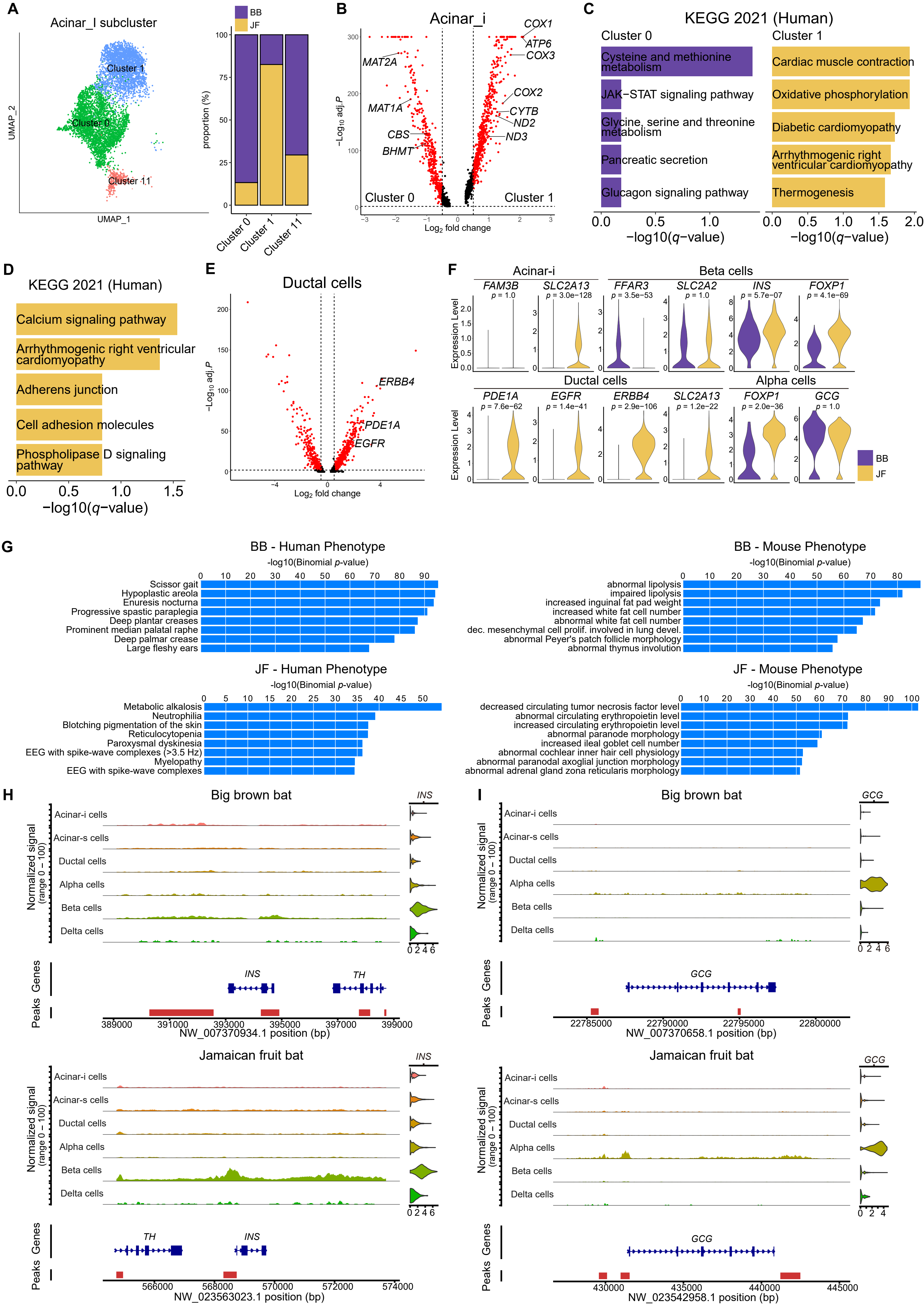

A
Acinar_I subcluster
Cluster 1
Cluster 0
Cluster 11
UMAP_2
UMAP_1
BB
JF
proportion (%)
Cluster 0
Cluster 1
Cluster 11
B
Acinar_i
COX1
ATP6
COX3
MAT2A
COX2
MAT1A
CYTB
ND2
CBS
ND3
BHMT
Cluster 0
Cluster 1
−Log10 adj.P
Log2 fold change
C
KEGG 2021 (Human)
Cluster 0
Cysteine and methionine metabolism
JAK−STAT signaling pathway
Glycine, serine and threonine metabolism
Pancreatic secretion
Glucagon signaling pathway
−log10(q−value)
Cluster 1
Cardiac muscle contraction
Oxidative phosphorylation
Diabetic cardiomyopathy
Arrhythmogenic right ventricular cardiomyopathy
Thermogenesis
−log10(q−value)
D
KEGG 2021 (Human)
Calcium signaling pathway
Arrhythmogenic right ventricular cardiomyopathy
Adherens junction
Cell adhesion molecules
Phospholipase D signaling pathway
−log10(q−value)
E
Ductal cells
ERBB4
PDE1A
EGFR
−Log10 adj.P
Log2 fold change
F
Acinar-i
Beta cells
FAM3B p = 1.0
SLC2A13 p = 3.0e−128
FFAR3 p = 3.5e−53
SLC2A2 p = 1.0
INS p = 5.7e−07
FOXP1 p = 4.1e−69
Expression Level
Ductal cells
Alpha cells
PDE1A p = 7.6e−62
EGFR p = 1.4e−41
ERBB4 p = 2.9e−106
SLC2A13 p = 1.2e−22
FOXP1 p = 2.0e−36
GCG p = 1.0
BB
JF
G
BB - Human Phenotype
-log10(Binomial p-value)
Scissor gait
Hypoplastic areola
Enuresis nocturna
Progressive spastic paraplegia
Deep plantar creases
Prominent median palatal raphe
Deep palmar crease
Large fleshy ears
BB - Mouse Phenotype
-log10(Binomial p-value)
abnormal lipolysis
impaired lipolysis
increased inguinal fat pad weight
increased white fat cell number
abnormal white fat cell number
dec. mesenchymal cell prolif. involved in lung devel.
abnormal Peyer's patch follicle morphology
abnormal thymus involution
JF - Human Phenotype
-log10(Binomial p-value)
Metabolic alkalosis
Neutrophilia
Blotching pigmentation of the skin
Reticulocytopenia
Paroxysmal dyskinesia
EEG with spike-wave complexes (>3.5 Hz)
Myelopathy
EEG with spike-wave complexes
JF - Mouse Phenotype
-log10(Binomial p-value)
decreased circulating tumor necrosis factor level
abnormal circulating erythropoietin level
increased circulating erythropoietin level
abnormal paranode morphology
increased ileal goblet cell number
abnormal cochlear inner hair cell physiology
abnormal paranodal axoglial junction morphology
abnormal adrenal gland zona reticularis morphology
H
Big brown bat
INS
Acinar-i cells
Acinar-s cells
Ductal cells
Alpha cells
Beta cells
Delta cells
Normalized signal (range 0 − 100)
Genes
Peaks
INS
TH
NW_007370934.1 position (bp)
Jamaican fruit bat
INS
TH
INS
NW_023563023.1 position (bp)
I
Big brown bat
GCG
Acinar-i cells
Acinar-s cells
Ductal cells
Alpha cells
Beta cells
Delta cells
Normalized signal (range 0 − 100)
Genes
Peaks
GCG
NW_007370658.1 position (bp)
Jamaican fruit bat
GCG
GCG
NW_023542958.1 position (bp)

Supplementary Fig.11: scRNA-seq and scATAC-seq analysis of bat pancreas.

a, (Left) UMAP of acinar-i subclusters. (Right) Proportion of species in each acinar-i subcluster. b, Volcano plot showing differentially expressed genes between acinar-i subcluster 0 (big brown (BB) bat-enriched) and subcluster 1 (Jamaican fruit (JF) bat-enriched). c, Bar plots showing KEGG Human 2021 pathways enriched in acinar-i subcluster 0 (big brown (BB) bat-enriched) and subcluster 1. *Q*-values calculated with one-sided Fisher's exact test and corrected with the Benjamini-Hochberg method. d, Bar plots showing KEGG Human 2021 pathways enriched in Jamaican fruit (JF) bat ductal cells. *Q*-values calculated with one-sided Fisher's exact test and corrected with the Benjamini-Hochberg method. e, Volcano plot showing differentially expressed genes between species in ductal cells. f, Violin plots of differentially expressed genes, except *FAM3B, SLC2A2*, and *GCG*, in bat pancreas cells. *P*-values calculated with two-sided Wilcoxon rank-sum test and the Bonferroni correction. g, Bar plots showing GREAT human and mouse phenotypes enriched in big brown (BB) bat and Jamaican fruit (JF) bat pancreases. h, scATAC-seq coverage plots of INS in bat pancreases. SCTransform-normalized expression plot visualized on the right by cell-type. i, scATAC-seq coverage plots of *GCG* in bat pancreases. SCTransform-normalized expression plot visualized on the right by cell-type. Jamaican fruit bat is depicted as JF and big brown bat as BB in the various panels. Source data are provided as a **Source Data** file.

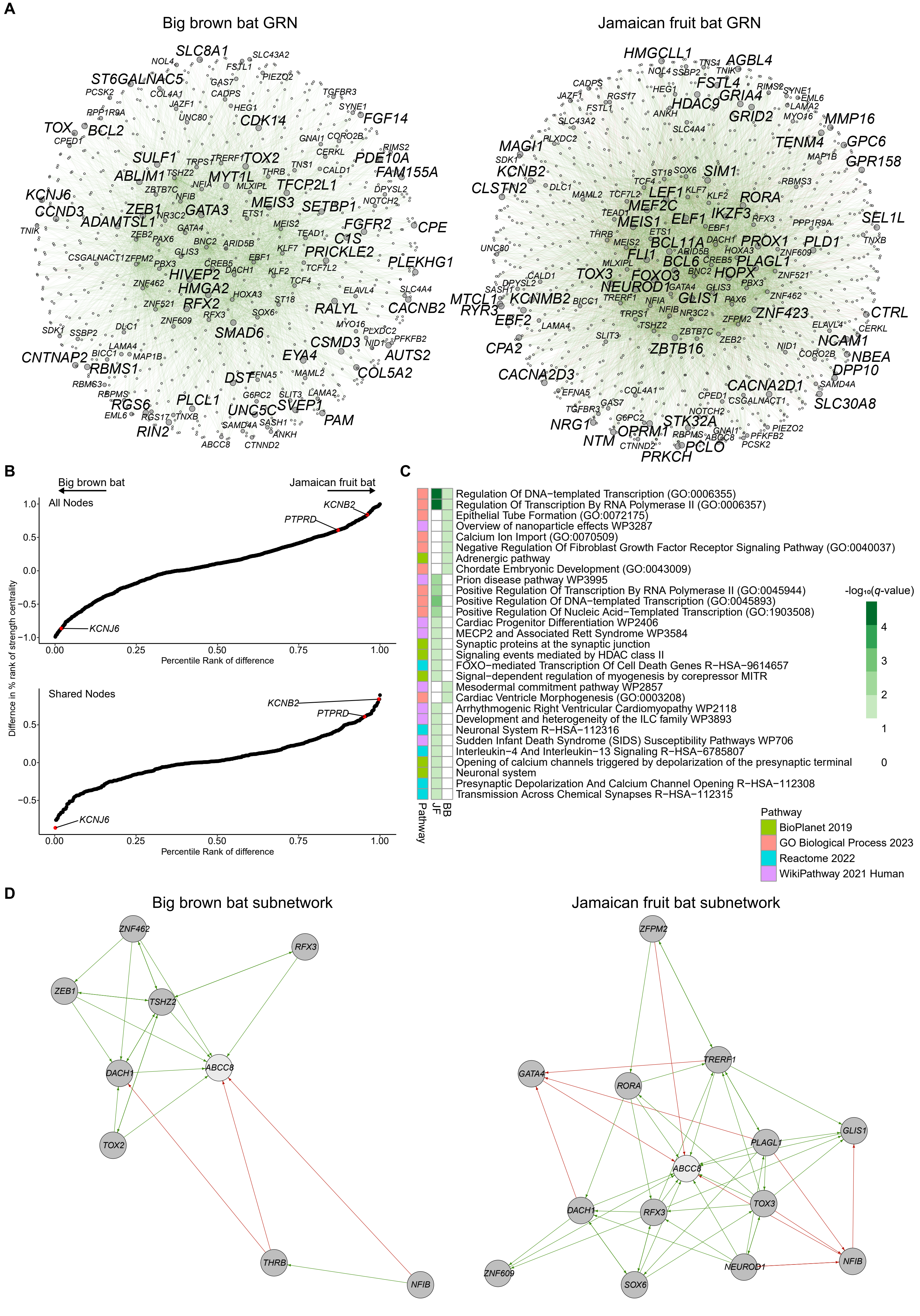
A
Big brown bat GRN
Jamaican fruit bat GRN
SLC8A1
ST6GALNAC5
CDK14
FGF14
TOX
BCL2
SULF1
TOX2
PDE10A
FAM155A
ABLIM1
MYT1L
TFCP2L1
KCNJ6
CCND3
ZEB1
GATA3
MEIS3
SETBP1
ADAMTSL1
FGFR2
CPE
C1S
PRICKLE2
PLEKHG1
HIVEP2
HMGA2
RFX2
RALYL
CACNB2
SMAD6
CSMD3
CNTNAP2
RBMS1
EYA4
AUTS2
COL5A2
DST
PLCL1
RGS6
UNC5C
SVEP1
PAM
RIN2
HMGCLL1
AGBL4
FSTL4
GRIA4
HDAC9
GRID2
MMP16
TENM4
GPC6
GPR158
MAGI1
KCNB2
SIM1
CLSTN2
LEF1
MEF2C
RORA
IKZF3
MEIS1
ELF1
SEL1L
BCL11A
PROX1
PLD1
FLI1
BCL6
PLAGL1
TOX3
FOXO3
HOPX
NEUROD1
GLIS1
MTCL1
KCNMB2
RYR3
ZNF423
CTRL
EBF2
ZBTB16
NCAM1
NBEA
CPA2
DPP10
CACNA2D3
CACNA2D1
SLC30A8
STK32A
NRG1
OPRM1
NTM
PCLO
PRKCH
B
Big brown bat
Jamaican fruit bat
All Nodes
KCNB2
PTPRD
KCNJ6
Percentile Rank of difference
Shared Nodes
KCNB2
PTPRD
KCNJ6
Percentile Rank of difference
Differnce in % rank of strength centrality
C
Regulation Of DNA−templated Transcription (GO:0006355)
Regulation Of Transcription By RNA Polymerase II (GO:0006357)
Epithelial Tube Formation (GO:0072175)
Overview of nanoparticle effects WP3287
Calcium Ion Import (GO:0070509)
Negative Regulation Of Fibroblast Growth Factor Receptor Signaling Pathway (GO:0040037)
Adrenergic pathway
Chordate Embryonic Development (GO:0043009)
Prion disease pathway WP3995
Positive Regulation Of Transcription By RNA Polymerase II (GO:0045944)
Positive Regulation Of DNA−templated Transcription (GO:0045893)
Positive Regulation Of Nucleic Acid−Templated Transcription (GO:1903508)
Cardiac Progenitor Differentiation WP2406
MECP2 and Associated Rett Syndrome WP3584
Synaptic proteins at the synaptic junction
Signaling events mediated by HDAC class II
FOXO−mediated Transcription Of Cell Death Genes R−HSA−9614657
Signal−dependent regulation of myogenesis by corepressor MITR
Mesodermal commitment pathway WP2857
Cardiac Ventricle Morphogenesis (GO:0003208)
Arrhythmogenic Right Ventricular Cardiomyopathy WP2118
Development and heterogeneity of the ILC family WP3893
Neuronal System R−HSA−112316
Sudden Infant Death Syndrome (SIDS) Susceptibility Pathways WP706
Interleukin−4 And Interleukin−13 Signaling R−HSA−6785807
Opening of calcium channels triggered by depolarization of the presynaptic terminal
Neuronal system
Presynaptic Depolarization And Calcium Channel Opening R−HSA−112308
Transmission Across Chemical Synapses R−HSA−112315
Pathway
JF
BB
-log₁₀(q-value)
Pathway
BioPlanet 2019
GO Biological Process 2023
Reactome 2022
WikiPathway 2021 Human
D
Big brown bat subnetwork
Jamaican fruit bat subnetwork
ZNF462
RFX3
ZEB1
TSHZ2
DACH1
ABCC8
TOX2
THRB
NFIB
ZFPM2
TRERF1
GATA4
RORA
GLIS1
PLAGL1
ABCC8
DACH1
RFX3
TOX3
ZNF609
SOX6
NEUROD1
NFIB

Supplementary Fig.12: Multi-omics GRN analyses of bat pancreases.

a, big brown (BB) bat and Jamaican fruit (JF) bat GRNs determined by Pando[5]. b, Scatter plots of the percentile rank of species difference by the difference in percentile rank of strength centrality for all nodes (top) and shared nodes (bottom). c, Heatmap of enriched pathways in each species GRN. *Q*-values calculated with one-sided Fisher's exact test and corrected with the Benjamini-Hochberg method. d, *ABCC8* subnetworks in each species. Jamaican fruit bat is depicted as JF and big brown bat as BB in the various panels. Source data are provided as a **Source Data** file.

Supplementary Table 1. Immunofluorescence antibodies and epitope matching to bats.

Gene	Thermo Scientific Antibody Catalog #	Big brown bat NCBI protein ID	Big brown bat NCBI Protein Blast % Identity	Jamaican fruit bat NCBI protein ID	Jamaican fruit bat NCBI Protein Blast % Identity
SLC12A1	18970-1-AP	XP_008141339.1	87.01	XP_037012367.1	86.36
AQP2	PA5-78808	XP_008138879.1	100	XP_036992308.1	100
SLC26A4	PA5-115911	XP_008148295.1	85.19	XP_037006378.1	96.3
INS	15848-1-AP	XP_008158493.1	81.82	XP_037012244.1	86.36
GCG	15954-1-AP	XP_027996892.1	78.33	XP_036996293.1	93.33

Supplementary References

1. Stuart, T., Srivastava, A., Madad, S., Lareau, C. A. & Satija, R. Single-cell chromatin state analysis with Signac. *Nat. Methods* **18**, 1333–1341 (2021).
2. Kirilenko, B. M. *et al.* Integrating gene annotation with orthology inference at scale. *Science* **380**, eabn3107 (2023).
3. Tang, H. *et al.* Synteny and collinearity in plant genomes. *Science* **320**, 486–488 (2008).
4. Hao, Y. *et al.* Integrated analysis of multimodal single-cell data. *Cell* **184**, 3573–3587.e29 (2021).
5. Fleck, J. S. *et al.* Inferring and perturbing cell fate regulomes in human brain organoids. *Nature* 1–8 (2022).
