# Supplementary material for: Integrative single-cell characterization of a frugivorous and an insectivorous bat kidney and pancreas
Source: Nat Commun. 2024 Jan 9;15:12. doi: 10.1038/s41467-023-44186-y (PMC10776631; doi:10.1038/s41467-023-44186-y)
Supplement: Supplementary file 4 — Reporting Summary [file 41467_2023_44186_MOESM4_ESM.pdf]

nature portfolio

Corresponding author(s): Martin Hemberg & Nadav Ahituv

Last updated by author(s): 10/25/2023

Reporting Summary

Nature Portfolio wishes to improve the reproducibility of the work that we publish. This form provides structure for consistency and transparency in reporting. For further information on Nature Portfolio policies, see our Editorial Policies and the Editorial Policy Checklist.

Please do not complete any field with "not applicable" or n/a. Refer to the help text for what text to use if an item is not relevant to your study.
For final submission: please carefully check your responses for accuracy; you will not be able to make changes later.

Statistics

For all statistical analyses, confirm that the following items are present in the figure legend, table legend, main text, or Methods section.

n/a	Confirmed	
☐	☒	The exact sample size (*n*) for each experimental group/condition, given as a discrete number and unit of measurement
☐	☒	A statement on whether measurements were taken from distinct samples or whether the same sample was measured repeatedly
☐	☒	The statistical test(s) used AND whether they are one- or two-sided<br>*Only common tests should be described solely by name; describe more complex techniques in the Methods section.*
☐	☒	A description of all covariates tested
☐	☒	A description of any assumptions or corrections, such as tests of normality and adjustment for multiple comparisons
☐	☒	A full description of the statistical parameters including central tendency (e.g. means) or other basic estimates (e.g. regression coefficient) AND variation (e.g. standard deviation) or associated estimates of uncertainty (e.g. confidence intervals)
☐	☒	For null hypothesis testing, the test statistic (e.g. *F*, *t*, *r*) with confidence intervals, effect sizes, degrees of freedom and *P* value noted<br>*Give P values as exact values whenever suitable.*
☒	☐	For Bayesian analysis, information on the choice of priors and Markov chain Monte Carlo settings
☒	☐	For hierarchical and complex designs, identification of the appropriate level for tests and full reporting of outcomes
☒	☐	Estimates of effect sizes (e.g. Cohen's *d*, Pearson's *r*), indicating how they were calculated

Our web collection on statistics for biologists contains articles on many of the points above.

Software and code

Policy information about availability of computer code

Data collection	No software was used for data collection.
Data analysis	Multiome_bat (Codes): https://doi.org/10.5281/zenodo.8254234, GetOrganelle (v1.7.5), MITOS (web server: http://mitos.bioinf.uni-leipzig.de/index.py), Orthofinder (v2.5.4), GenomicRanges (v1.50.2), Seurat (v4.0), Harmony (v0.1.1), SCTransform (v0.3.3), Signac (v1.8.0), Ggplot2 (v3.4.0), CNA (v0.0.99), VCD (v1.4.10), Azimuth (v0.4.6), Pheatmap (v1.0.12), CellProfiler (v4.2.4), EnrichR (v3.0), TOGA (v1.13), JCVI - MCscan (v1.3.6), MACS2 (v2.2.5), GREAT (v4.0.4, web software: http://great.stanford.edu/public/html/index.php), Lastz (v1.04), UCSC Genome Bioinformatics Group tools (v384) MEMES – AME/FIMO (v1.6.0), BSgenome (v1.68.0), Pando (v1.0.5), Igraph (v1.5.0)

For manuscripts utilizing custom algorithms or software that are central to the research but not yet described in published literature, software must be made available to editors and reviewers. We strongly encourage code deposition in a community repository (e.g. GitHub). See the Nature Portfolio guidelines for submitting code & software for further information.

Data

Policy information about availability of data

All manuscripts must include a data availability statement. This statement should provide the following information, where applicable:

- Accession codes, unique identifiers, or web links for publicly available datasets
- A description of any restrictions on data availability
- For clinical datasets or third party data, please ensure that the statement adheres to our policy

Raw 10X multiome sequencing data generated in this study have been deposited at NCBI BioProject under accession code PRJNA916224 [https://www.ncbi.nlm.nih.gov/bioproject/PRJNA916224]. Big brown bat mitochondrial genome nucleotide sequence data reported are available in the Third Party Annotation Section of the DDBJ/ENA/GenBank databases under the accession number TPA: BK063052 [https://www.ncbi.nlm.nih.gov/nuccore/BK063052/]. Human genome (*hg38*) sequence used for Jamaican fruit bat pairwise alignment can be found at GenBank:GCA_000001405.19 [https://www.ncbi.nlm.nih.gov/datasets/genome/GCA_000001405.19/]. Mouse genome (*mm39*) and human genome (*hg38*) sequences used for MCscan can be found at GenBank: GCA_000001635.9 [https://www.ncbi.nlm.nih.gov/datasets/genome/GCA_000001635.9/] and GCA_000001405.29 [https://www.ncbi.nlm.nih.gov/datasets/genome/GCA_000001405.29/]. All data are available in the main text or the supplementary materials.

Research involving human participants, their data, or biological material

Policy information about studies with human participants or human data. See also policy information about sex, gender (identity/presentation), and sexual orientation and race, ethnicity and racism.

Reporting on sex and gender	Not applicable.
Reporting on race, ethnicity, or other socially relevant groupings	Not applicable.
Population characteristics	Not applicable.
Recruitment	Not applicable.
Ethics oversight	Not applicable.

Note that full information on the approval of the study protocol must also be provided in the manuscript.

Field-specific reporting

Please select the one below that is the best fit for your research. If you are not sure, read the appropriate sections before making your selection.

[X] Life sciences [ ] Behavioural & social sciences [ ] Ecological, evolutionary & environmental sciences

For a reference copy of the document with all sections, see nature.com/documents/nr-reporting-summary-flat.pdf

Life sciences study design

All studies must disclose on these points even when the disclosure is negative.

Sample size	4 kidneys and 3 pancreases of adult male A. jamaicensis, 4 kidneys and 4 pancreases of adult male E. fuscus for joint scATAC/RNA-seq.<br>3 kidneys and pancreases of adult male E. fuscus, 2 kidneys and pancreases of adult male A. jamaicensis, 1 kidney and pancreas of adult female A. jamaicensis used for immunofluorescence experiments.
Data exclusions	None
Replication	2 fasted and 2 fed for A. jamaicensis and E. fuscus kidneys and pancreases, 1 fed and 2 fasted for A. jamaicensis pancreas
Randomization	None
Blinding	None

Behavioural & social sciences study design

All studies must disclose on these points even when the disclosure is negative.

Study description	
Research sample	
Sampling strategy	
Data collection	
Timing	
Data exclusions	
Non-participation	
Randomization	

Ecological, evolutionary & environmental sciences study design

All studies must disclose on these points even when the disclosure is negative.

Study description	
Research sample	
Sampling strategy	
Data collection	
Timing and spatial scale	
Data exclusions	
Reproducibility	
Randomization	
Blinding	

Did the study involve field work? ☐ Yes ☐ No

Field work, collection and transport

Field conditions	
Location	
Access & import/export	
Disturbance	

Reporting for specific materials, systems and methods

We require information from authors about some types of materials, experimental systems and methods used in many studies. Here, indicate whether each material, system or method listed is relevant to your study. If you are not sure if a list item applies to your research, read the appropriate section before selecting a response.

Materials & experimental systems

n/a	Involved in the study	
☐	☒	Antibodies
☒	☐	Eukaryotic cell lines
☒	☐	Palaeontology and archaeology
☐	☒	Animals and other organisms
☒	☐	Clinical data
☒	☐	Dual use research of concern
☒	☐	Plants

Methods

n/a	Involved in the study	
☒	☐	ChIP-seq
☒	☐	Flow cytometry
☒	☐	MRI-based neuroimaging

Antibodies

Antibodies used

Thermo Scientific Antibody Catalog #s: 18970-1-AP (Lot: 00045810; 1:100), PA5-78808 (Lot: XK3757771;1:100), PA5-115911 (Lot: XK3769784; 1:100), 15848-1-AP (Lot: 00060092; 1:500), 15954-1-AP (Lot: 00026437;1:500)

Validation

18970-1-AP: Thermo Scientific tested in IHC (F); published species: human & mouse; 27 references
PA5-78808: Thermo Scientific Advanced Verification; Thermo Scientific tested in IHC (F)
PA5-115911: Thermo Scientific tested in IF
15848-1-AP: Thermo Scientific tested in IF; published species: human, mouse, rat; 68 references
15954-1-AP: Thermo Scientific tested in IF; published species: human, mouse, rat; 31 references

Eukaryotic cell lines

Policy information about [cell lines and Sex and Gender in Research]

Cell line source(s)

Authentication

Mycoplasma contamination

Commonly misidentified lines
(See [ICLAC] register)

Palaeontology and Archaeology

Specimen provenance

Specimen deposition

Dating methods

☐ Tick this box to confirm that the raw and calibrated dates are available in the paper or in Supplementary Information.

Ethics oversight

Note that full information on the approval of the study protocol must also be provided in the manuscript.

Animals and other research organisms

Policy information about [studies involving animals; ARRIVE guidelines] recommended for reporting animal research, and [Sex and Gender in Research]

Laboratory animals

Four adult male *Artibeus jamaicensis* fruit-eating bats (Colorado State University [CSU]) and five adult male *Eptesicus fuscus* insect-eating bats (6-7 years old; Northeast Ohio Medical University [NEOMED]). All Jamaican fruit bats were fasted overnight during their night cycle (approximately 12 hours), and "fasted" bats were removed from their enclosures before feeding for euthanasia. "Fed" Jamaican fruit bats were provided unlimited cantaloupe and banana and were euthanized 30 minutes later. Tissues were harvested immediately for flash-freezing (May 2021) and stored at -80 Celsius until nuclei isolation. Because big brown bats feed on mealworms *(Tenebrio molitor*), we increased the fruit content of these mealworms to create a fruit "treatment" for insect-eating bats. These mealworms were fed a modified fruit diet for four days of sweet potatoes, apples, mango, and cane sugar. "Fasted" big brown bats were removed from their enclosures before feeding for euthanasia, and three "fed" big brown bats were fed an unlimited supply of fruit-enriched mealworms and were euthanized 30 minutes later. Tissues were harvested immediately for flash-freezing (all big brown bats were euthanized before the winter (September 2020) to exclude hibernation effects) and stored at -80 Celsius until nuclei isolation.

Wild animals

The individual female Artibeus jamaicensis sampled (field number BZ701, voucher number AMNH.Mammalogy.281145) was caught in a ground-level mist net set near the High Temple at Lamanai (17.76750 N, 88.65207 W) on 17 November 2021. The bat was subjected to minimal handling after capture, and it was held in a clean cloth bag after capture as per best practices for field containment of bats. After species identification, the individual was euthanized humanely by isoflurane inhalation the same night it was captured.

Reporting on sex

all male with exception of one female A. jamaicensis used for immunofluorescence experiments

Field-collected samples

The individual female Artibeus jamaicensis sampled (field number BZ701, voucher number AMNH.Mammalogy.281145) was caught in a ground-level mist net set near the High Temple at Lamanai (17.76750 N, 88.65207 W) on 17 November 2021. The bat was subjected to minimal handling after capture, and it was held in a clean cloth bag after capture as per best practices for field containment of bats. After species identification, the individual was euthanized humanely by isoflurane inhalation the same night it was captured.

Ethics oversight

NEOMED IACUC #20-06-721, CSU IACUC #1034, AMNH IACUC-20191212, Belize Institute of Archaeology Permit IA/S/5/6/21(01), Belize Forest Department Permit FD/WL/1/21(16), Belize Forest Department permit FD/WL/7/22(08)

Note that full information on the approval of the study protocol must also be provided in the manuscript.

Clinical data

Policy information about [clinical studies]

All manuscripts should comply with the ICMJE [guidelines for publication of clinical research] and a completed [CONSORT checklist] must be included with all submissions.

Clinical trial registration

Study protocol

Data collection

Outcomes

Dual use research of concern

Policy information about [dual use research of concern]

Hazards

Could the accidental, deliberate or reckless misuse of agents or technologies generated in the work, or the application of information presented in the manuscript, pose a threat to:

No	Yes	
X	☐	Public health
X	☐	National security
X	☐	Crops and/or livestock
X	☐	Ecosystems
X	☐	Any other significant area

Experiments of concern

Does the work involve any of these experiments of concern:

No	Yes	
X	☐	Demonstrate how to render a vaccine ineffective
X	☐	Confer resistance to therapeutically useful antibiotics or antiviral agents
X	☐	Enhance the virulence of a pathogen or render a nonpathogen virulent
X	☐	Increase transmissibility of a pathogen
X	☐	Alter the host range of a pathogen
X	☐	Enable evasion of diagnostic/detection modalities
X	☐	Enable the weaponization of a biological agent or toxin
X	☐	Any other potentially harmful combination of experiments and agents

Plants

Seed stocks	
Novel plant genotypes	
Authentication	

ChIP-seq

Data deposition

☐ Confirm that both raw and final processed data have been deposited in a public database such as GEO.

☐ Confirm that you have deposited or provided access to graph files (e.g. BED files) for the called peaks.

Data access links<br>*May remain private before publication.*	
Files in database submission	
Genome browser session<br>(e.g. UCSC)	

Methodology

Replicates	
Sequencing depth	
Antibodies	
Peak calling parameters	
Data quality	
Software	

Flow Cytometry

Plots

Confirm that:

☐ The axis labels state the marker and fluorochrome used (e.g. CD4-FITC).

☐ The axis scales are clearly visible. Include numbers along axes only for bottom left plot of group (a 'group' is an analysis of identical markers).

☐ All plots are contour plots with outliers or pseudocolor plots.

☐ A numerical value for number of cells or percentage (with statistics) is provided.

Methodology

Sample preparation

Instrument

Software

Cell population abundance

Gating strategy

☐ Tick this box to confirm that a figure exemplifying the gating strategy is provided in the Supplementary Information.

Magnetic resonance imaging

Experimental design

Design type

Design specifications

Behavioral performance measures

Imaging type(s)

Field strength

Sequence & imaging parameters

Area of acquisition

Diffusion MRI ☐ Used ☐ Not used

Preprocessing

Preprocessing software

Normalization

Normalization template

Noise and artifact removal

Volume censoring

Statistical modeling & inference

Model type and settings

Effect(s) tested

Specify type of analysis: ☐ Whole brain ☐ ROI-based ☐ Both

Statistic type for inference

(See Eklund et al. 2016)

Correction

Models & analysis

n/a	Involved in the study
☐	☐ Functional and/or effective connectivity
☐	☐ Graph analysis
☐	☐ Multivariate modeling or predictive analysis

Functional and/or effective connectivity

Graph analysis

Multivariate modeling and predictive analysis

This checklist template is licensed under a Creative Commons Attribution 4.0 International License, which permits use, sharing, adaptation, distribution and reproduction in any medium or format, as long as you give appropriate credit to the original author(s) and the source, provide a link to the Creative Commons license, and indicate if changes were made. The images or other third party material in this article are included in the article's Creative Commons license, unless indicated otherwise in a credit line to the material. If material is not included in the article's Creative Commons license and your intended use is not permitted by statutory regulation or exceeds the permitted use, you will need to obtain permission directly from the copyright holder. To view a copy of this license, visit http://creativecommons.org/licenses/by/4.0/
